# PP27 Evidence Quality and HTA outcomes in Reappraisals For Drugs For Rare Diseases In Germany

**DOI:** 10.1017/S0266462324002009

**Published:** 2025-01-07

**Authors:** Lea Wiedmann, John Cairns, Ellen Nolte

## Abstract

**Introduction:**

Evidence on re-appraisals of health technologies in Germany is limited, and for rare disease treatments (RDTs) the Federal Joint Committee (GBA) uses different processes depending on whether the annual revenue threshold has been exceeded. We analyze RDTs for which an initial appraisal and a reappraisal were conducted to better understand processes used to determine the clinical benefit rating and their outcomes.

**Methods:**

We review appraisal documents for 55 RDT indications published between January 2011 and September 2023. We extract information for the change in the maturity of survival data, the type of evidence, the risk of bias, and the availability of additional evidence as proxies for evidence quality. Specifically, we review the reasons for conducting reappraisals; examine how evidence quality and the clinical benefit rating differed between initial appraisals and re-appraisals; and explore the association between evidence quality and the clinical benefit rating following reappraisal.

**Results:**

Most reappraisals were conducted because of exceeding the revenue threshold of EUR50 million (USD54 million) per year or reaching the review date when an initial decision was time limited. Almost all initial appraisals used the limited process, while the majority of reappraisals used the regular process. While nine out of 55 reappraisals achieved a higher benefit rating in reappraisals compared to initial appraisals, in 21 out of 55 reappraisals the benefit rating decreased. There was some evidence that reappraisals with accepted randomized controlled trial evidence were significantly more likely to achieve a higher clinical benefit rating.

**Conclusions:**

Our findings confirm that reasons and processes for completing reappraisals of RDTs in Germany differ. Moreover, the quality of the evidence submitted for both initial appraisals and reappraisals of RDTs was limited and achieving a high clinical benefit rating in reappraisals was rare.

